# What mechanisms drive uptake of family planning when integrated with childhood immunisation in Ethiopia? A realist evaluation

**DOI:** 10.1186/s12889-020-10114-8

**Published:** 2021-01-07

**Authors:** Shari Krishnaratne, Jessie K. Hamon, Jenna Hoyt, Tracey Chantler, Justine Landegger, Nathaly Spilotros, Shiferaw Dechasa Demissie, Siraj Mohammed, Jayne Webster

**Affiliations:** 1 Department of Disease Control, Faculty of Infectious and Tropical Diseases, London School of Hygiene & Tropical Medicine, London, UK; 2 Department of Global Health and Development, Faculty of Public Health and Policy, London School of Hygiene & Tropical Medicine, London, UK; 3 International Rescue Committee, New York, USA; 4 International Rescue Committee, Addis Ababa, Ethiopia

**Keywords:** Family planning, Immunisation, Integration, Realist evaluation

## Abstract

**Background:**

Maternal and child health are key priorities among the Sustainable Development Goals, which include a particular focus on reducing morbidity and mortality among women of reproductive age, newborns, and children under the age of five. Two components of maternal and child health are family planning (FP) and immunisation. Providing these services through an integrated delivery system could increase the uptake of vaccines and modern contraceptive methods (MCMs) particularly during the post-partum period.

**Methods:**

A realist evaluation was conducted in two woredas in Ethiopia to determine the key mechanisms and their triggers that drive successful implementation and service uptake of an intervention of integrated delivery of immunisations and FP. The methodological approach included the development of an initial programme theory and the selection of relevant, published implementation related theoretical frameworks to aid organisation and cumulation of findings. Data from 23 semi-structured interviews were then analysed to determine key empirical mechanisms and drivers and to test the initial programme theory. These mechanisms were mapped against published theoretical frameworks and a revised programme theory comprised of context-mechanism-outcome configurations was developed. A critique of theoretical frameworks for abstracting empirical mechanisms was also conducted.

**Results:**

Key contextual factors identified were: the use of trained Health Extension Workers (HEWs) to deliver FP services; a strong belief in values that challenged FP among religious leaders and community members; and a lack of support for FP from male partners based on religious values. Within these contexts, empirical mechanisms of acceptability, access, and adoption of innovations that drove decision making and intervention outcomes among health workers, religious leaders, and community members were identified to describe intervention implementation.

**Conclusions:**

Linking context and intervention components to the mechanisms they triggered helped explain the intervention outcomes, and more broadly how and for whom the intervention worked. Linking empirical mechanisms to constructs of implementation related theoretical frameworks provided a level of abstraction through which findings could be cumulated across time, space, and conditions by theorising middle-range mechanisms.

**Supplementary Information:**

The online version contains supplementary material available at 10.1186/s12889-020-10114-8.

## Background

Integrated approaches to health service delivery in low- and middle-income countries have steadily increased in scope and scale over the last decade. Evidence suggests that integrating health services can effectively address accessibility issues created or perpetuated by disjointed health services and fragmented funding [1]. For instance, a systematic review of strategies for integrating health services suggested that integration can lead to more efficient service delivery and that by reducing the burden on those seeking health care, it may increase access to health services overall [1]. The review also recognised the potential of integration to over-burden health care workers (HCWs) leading to their burnout and reducing the quality of the care they deliver, and highlighted that integration does little to address pre-existing inequities.

Recently, the integration of essential services such as childhood immunisations and reproductive health services has garnered attention from policy makers, implementers and researchers, in light of the Sustainable Development Goal of reducing child mortality and improving maternal health [2]. However, to date, few studies have focused on the integration of FP services and childhood immunisation as an approach to increase uptake of modern contraceptive methods (MCMs) and to improve maternal and child health [3–9], despite evidence linking short interpregnancy intervals to adverse health outcomes for mothers, infants, and children. Such adverse outcomes include: maternal death, third-trimester bleeding, and anaemia in mothers, as well as preterm birth and low birthweight in infants [10–12].

An intervention that integrated the delivery of FP with childhood immunisation services in Assosa and Bambasi woredas (districts), in the Benishangul-Gumuz Regional State (BGRS) of Ethiopia, offered the opportunity to contribute to this evidence base. The intervention was implemented in all 114 government health posts of Assosa and Bambasi woredas with support from the International Rescue Committee between January 2016 and May 2018. It aimed to strengthen FP, and childhood immunisation services, refine referral pathways for both services, improve immunisation monitoring, and build HCW capacity.

The intervention included: 1) training and mentoring of facility-based health extension workers (HEWs) on FP counselling and short-acting MCM provision and implant insertions which included the use of anatomic models for the practice of implant insertions and clinical coaching of insertions in the community; 2) expanding the range of MCM options available at health posts to include implant insertions (but not removals); 3) designing a job aid to support HEWs to improve immunisation defaulter tracing; and 4) developing community engagement strategies that involved community leaders and kebele command posts, which reviewed health post performance data and helped HEWs troubleshoot problems they encountered.

The intervention was mainly delivered by HEWs who provided FP counselling and aministered MCMs during household visits and at health or outreach posts (in particularly large woredas). Specifically, post-partum women were counselled on FP during the 1st, 3rd, and 7th day post-natal checks and MCMs were provided alongside the child’s ‘45 day immunisation’ or at a later date during a household visit or at a health or outreach post. The 45 day immunisation takes place 6 weeks after birth and is the first round of immunisations including Penta1 (DTP-HepB1-Hib1,OPV1,PCV1, Rota1). The provision of contraceptive implants by HEWs was a central component of the intervention. Though HEWs were trained to insert implants, national policy at the time of the intervention stipulated that implant removals should not be performed by HEWs, therefore this task was not included in the intervention [13]. Other HCWs, such as nurses and midwives, also played a key role in delivering the intervention by providing clinical coaching to HEWs and accepting referral clients from health posts at larger health facilities. According to monitoring data, between January 2017 and May 2018, the proportion of women who brought a child for immunisation at least once and who received an MCM was 63.0% (4260/6764). These data reflect the communities that were exposed to the intervention for 12 to 17 months. A total of 25,058 FP acceptors were recorded in Assosa and Bambasi between January 2017 and May 2018, of which 7945 (31.7%) were new FP acceptors and 17,113 (68.3%) were repeat acceptors.

We conducted an evaluation of the intervention with the aim of determing why (or why not) and for whom the intervention worked, by identifying and interrogating the mechanisms that drove implementation of the intervention.

## Methods

### Methodological approach

Process-focused, theory based realist evaluation presents a useful framework when seeking to answer questions of what works, for whom, and under what circumstances. A central tenet of realist evaluation is that interventions work, or do not work, based upon the decisions that actors make in response to available resources. These decisions constitute mechanisms which are triggered in some contexts and not in others. We explored this relationship between context, mechanism, resources/intervention, and resulting oucome(s) using the context-mechanism-outcome (CMO) configuration of realist evaluation [14]. Where a CMO relates to a specific category of actor, a context-actor-mechanism-outcome (CAMO) is useful, and where the CMO relates to an intervention or component of an intervention, then a context-intervention-actor-mechanism-outcomes (CIAMO) configuration is better able to specify what works, for whom, why and where.

**Fig. 1:**
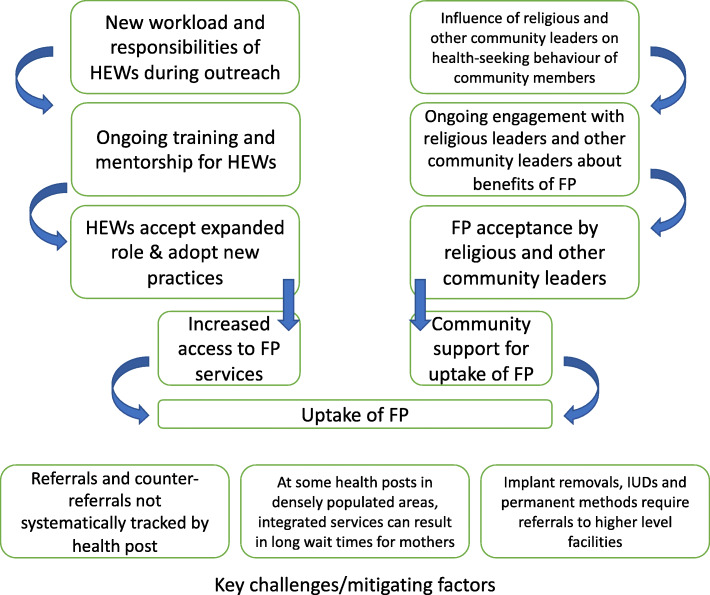
Initial programme theory

We used two further theory based approaches alongside the CMO/CAMO/CIAMO heuristic in our interrogation of the uptake of FP within the integrated service delivery model. The first of these was the development of an initial programme theory (Fig. 1). Discussions were held with intervention designers and implementers in a workshop 15 months into the implementation of the intervention. This exercise focussed on implementers’ understanding of how the intervention and its components were expected to work, how they were currently perceived to be working, and how CMO/CAMO/CIAMO configurations could be used to determine and explain factors enabling or hindering the intervention. In constructing the initial programme theory we were able to elucidate factors that intervention designers and implementers perceived as the major drivers of the intervention [15]. These were adherence to clinical and counselling guidelines among HEWs, and community and religious leader support for FP. The initial programme theory also described potential barriers and mitigating factors to intervention implementation including the lack of tracking for referrals to higher level facilities for FP made by HEWs at health posts, long wait times at health posts in densely populated communities, and the lack of HEW training on implant removals. This initial programme theory was used in developing themes for interview guides and in identifying stakeholders for empirical interviews used to develop CMO/CAMO/CIAMOs.

Our second approach was to map our findings against the constructs of implementation related theoretical frameworks. Our reasons for using this approach hinge upon two methodological axes which are that the major challenge for evaluation is the cumulation of findings across time, space and conditions [14] and that for realist evaluation, such generalisation or transferability of findings occurs through abstraction. Abstraction is achieved through linking to theories including those from cognitive psychology, and behavioural science [16] and more recently also including a range of theories relating to behaviour change in health systems [17]. We assumed that the use of the constructs of implementation related frameworks rather than broader behavioural theories, for example, would provide more insight on mechanisms driving implementation outcomes and opportunites for cumulation of findings across evaluations.

We considered several theories and concluded that our initial programme theory had best fit with constructs of three theoretical frameworks. These were acceptability of FP and MCMs by both health workers and community members [18], adoption and diffusion of innovations particularly with respect to health workers [19, 20], and access by women [21]. The constructs described by these frameworks align with the concept of mechanisms within realist evaluation as they describe factors that drive or lead to decision making among different actors. By mapping our findings against these constructs, we aimed to identify transferrable theories which could be used by implementers in similar contexts. Sekhon et al describe acceptability using seven constructs in the Theoretical Framework of Acceptability (TFA): affective attitude, burden, perceived effectiveness, ethicality, intervention coherence, opportunity costs, and self-efficacy [18]. Rogers’ diffusion (and adoption) of innovations framework consists of five contructs: relative advantage, compatibility, trialability, observability and complexity [20]. Finally, Penchansky and Thomas describe access using five constructs: availability, accessibility, accommodation, affordability and acceptability [21]. We linked the empirical mechanisms identified in the data to a construct(s) of the frameworks and critiqued the potential of this approach as an aid to cumulation of findings across studies.

### Study site

BGRS is one of nine regional states in Ethiopia. It is predominantly rural and consists of twenty woredas (districts) and 398 kebeles (smallest administrative unit) [22]. Assosa and Bambasi are part of BGRS and encompass 74 and 40 kebeles respectively. Within BGRS, there are five native Ethnic groups (Bertha, Gumuz, Mao, Komo and Shenash) and other dwellers (predominantly Oromo and Amhara). The region has relatively low levels of literacy (60.9% of women and 30.3% of men are illiterate), and high religiosity, with Islam and Orthodox Christianity being the predominant religions (51.3 and 28.2% respectively) [22, 23]. At the time of the 2016 Demographic Health Survey (DHS), full immunisation coverage in BGRS was 57.4% compared to nearly 89.2% in the capital of Addis Ababa and a national average of 38.3% [23]. The proportion of women aged 15 to 49 using any FP method in BGRS was 28.5% compared to 55.9% in Addis Ababa. Knowledge about FP was only slighty lower in BGRS compared to Addis Ababa (97.6% vs 100.0% respectively). There was also more male involvement in decision-making about FP in BGRS compared to Addis Ababa: 9.8% compared to 2.4% of women reported that their male partner was the main decision maker about FP, while 14.4% compared to 25% of women said they made the decisions about FP. However, the majority of respondents said that decision making was done jointly (75.9% vs 72.2%) [23].

In BGRS, the Health Extension Programme (HEP) plays a key role in health service delivery by providing primary health services at health posts in rural communities. It was adopted by the government of Ethiopia in 2003 to achieve universal health coverage among rural populations by 2009 [24]. The HEP is driven by model families, the Health Development Army (HDA) and HEWs [24]. Model families are male and female headed households that have received specific training on the HEP and that follow best practices for health and hygiene. They serve as role models within the community [24]. The HDA is an organised community based movement aimed at improving health sector capacity by engaging with communities and community leaders [24]. HEWs, commonly women, typically staff health posts in pairs and provide services such as community integrated management of childhood illness, immunisations, injectable contraceptives, implant insertions (but not removals), as well as basic curative services such as first aid and malaria treatment. HEWs are the lowest level health cadre in Ethiopia, usually with an education up to Grade 10, supplemented with a 1 year didactic and practical training in different health care packages. Among other responsibilities, HEWs conduct household visits and outreach activities and refer cases to health centers as needed.

### Empirical data collection and sampling

Semi-structured interviews (SSIs) with key stakeholders involved in the delivery and uptake of the intervention were conducted to identify contextual factors that triggered the mechanisms driving intervention outcomes. Purposive sampling was used for SSIs to select key stakeholders involved in, or with an interest in, the intervention including implementing partners, government officials, HEWs, and community leaders. Participants were selected to offer a range of perspectives and opinions of the intervention. HEWs selected were involved in the delivery of childhood immunisation and/or FP services and were from health posts where the intervention was perceived to be more, or less, well received based on project monitoring data.

An interview and discussion guide for SSIs was developed specifically for this study and was informed by the initial programme theory. Broad themes encompassed workload, socio-cultural norms, and healthcare access, and questions specific to particular participant groups and specific aspects of the intervention within the study context were included. This ensured that key issues captured within the initial programme theory were included in the interviews. Please see supplementary file S1 for the interview guide that was used. CMOs developed with the implementers were also included in interview and discussion guides [25]. Interviews were conducted in October 2017 and March 2018 in Amharic and Afan Oromo by local research assistants with guidance and oversight from a London School of Hygiene & Tropical Medicine researcher and an implementation supervisor. All interviews were recorded, transcribed *verbatim* and then translated into English.

### Data management and analysis

Translated transcripts were imported into NVivo 11.2 for coding and analysis. Quotes were anonymized, but the type of respondent attributable to each quote was retained to aid analyses. Key themes were identified based on the interview guides and supported by quotes from interview transcripts. Coding and analysis was based on an intial framework of: interventions; actors; context; mechanisms; outcomes and initial CAMO and CIAMO configurations. These categories were populated inductively with themes and sub-themes as they were identified from the data.

We developed CMO/CAMO/CIAMO configurations from the analysis of stakeholder interviews. Overarching contexts were identified as well as contextual and intervention triggers for specific mechanisms driving outcomes. The outcomes included in the CAMOs and CIAMOs were both outputs and outcomes. We then linked the identified mechanisms with constructs of the acceptability, adoption and diffusion of innovations, and access frameworks. Finally, we used the CMO/CAMO/CIAMO configurations to construct a revised programme theory.

## Results

Twenty-three stakeholders SSIs were conducted (Table 1).

**Table 1:** Participants in stakeholder interviews (SSIs)

Type of Participant	Number of participants
Religious Leader	1 SSI
Health Extension worker (HEW)	4 SSIs
Health Development Army/Leader (HDAL)	2 SSIs & 2 SSIs
Mothers (FP user)	1 SSI
Nurse	2 SSIs
Health professional	1 SSI
HEW supervisor	1 SSI
Woreda level officers	5 SSI
Kebele leaders	3 SSIs
Group 1–5 leader	1 SSI

### Context-mechanism-outcome configurations

Nine mechanisms were identified from the analysis. Six of these mechanisms were a reaction to a component of the intervention within the prevailing context (CIAMOs) and three were a direct reaction to the prevailing context (CAMOs) (Table 2).

**Table 2:** Context-Intervention-Actor-Mechanism-Outcome (CIAMO) and Context-Actor-Mechanism-Outcome (CAMO) configurations and acceptability constructs

Context	Project interventions	Actor, mechanism, and outcome	Constructs of acceptability, diffusion of innovations, and/or accessibility
**CIAMO 1**: Healthcare delivery is conducted by HEWs at health posts and home (C)	EPI and FP services offered at the 45 day post-natal check (I)	HEWs (A) perceive a reduced work burden due to EPI/FP service integration (M) and therefore provide integrated services (O)	Burden, affective attitude, observability, relative advantage
	“*R: I strongly feel that having everything integrated is beneficial and actually makes my job easier. For example, when we go to vaccinate a child at 45 days we have to meet with the mother anyway and so that opportunity is used to also offer contraception. In my opinion this is a reduction of work rather than an increase.”* HEW_3		
**CIAMO 2**: Healthcare delivery is conducted by HEWs at health posts and home (C)	Ongoing training on EPI and FP integrated service delivery (I)	HEWs (A) feel that providing both services together has more impact’ (M) and therefore provide integrated services (O)	Perceived effectiveness, relative advantage, compatibility, observability
	“R: *Because this project has allowed me and other health workers to address vaccinations and family planning together as one package. Therefore, I feel our efforts have more of an impact than they did prior to the project. We are now seeing better outcomes because of its introduction.”* HEW_2		
**CIAMO 3**: FP delivery is conducted by HEWs (C)	HEWs given on-the job mentoring on implant insertion (I)	HEWs (A) feel confident in their ability to provide implants for women (M) and therefore provide integrated services including implants (O)	Self-efficacy, affective attitude, trialability
	“R: *Previously, the long-acting family planning was given at health centre level. Currently, it is given by the health extension workers after they take training …*. T*hey took the training but since they haven’t done this before, they may lack confidence. We overcame this by onsite mentorship with the presence of trained officer from the Woreda office, IRC and us. We made appointments with mothers to come and mentored the extension workers to practice giving the service while the team is there. Then we got in to the actual work after they practiced and started doing by themselves. Now it is good …*.” Regional level coordinator_1		
**CAMO 1**: HEWs are unable to remove implants (C)	No defined intervention (I)	HEWs (A) worry about not being able to remove implants (M) and therefore are limited in the FP services they can provide (O)	Self-efficacy
	“*R: I have only taken training with regarding to administering the contraceptives. I have not had training in removals. Removals are a bit of challenge here because none of us are currently carrying them out.”* Nurse_1		
**CIAMO 4**: Strong belief in religious values among religious leaders and within the community (C)	Analysis of religious text together with religious leaders (I)	Religious leaders (A) recognise that FP aligns with their religious values (M) and support the use of FP (O)	Ethicality, opportunity costs, compatibility,
	*“The religious leaders were first saying that family planning was Haram but since the project they had increase awareness and now are fully on board to point that they are teaching about family planning in the Mosque.” MCH Woreda officer_1*		
**CIAMO 5**: Religious leaders accept that FP aligns with religious values (C)	Religious leaders openly promote alignment of FP with religious principles (I)	Male partners (A) respect and trust the views of religious leaders (M) and support the use of FP (O)	Ethicality, opportunity costs, compatibility
	*“I did have a situation where the women wanted the contraception on the same day as the immunisation day but her husband, who was with her at the time did not want her to take any contraception … What I then did was go to their house together with another religious leader to educate the husband about the benefits of family planning. To my surprise he actually agreed for his wife to have the 3 year implant.” HEW_1*		
**CAMO 2**: Supportive community environment for FP (C)	No defined intervention (I)	Women (A) feel supported by their partners and the wider community when making decisions about FP (M) and choose to take up an MCM (O)	Self-efficacy
	*I: What is your husband’s opinion regarding this program? R: He says nothing. We have agreed. There is no problem. I: What did he say when you first start it? R: After we have agreed, he asked me how long it was for and I told him that the 3 years is better. I explained to him that after our children grow with good health and clothes, I will then remove it and have another child. I: Did he agree on that? R: Yes, we have agreed.” Woman user_2*		
**CIAMO 6**: Women want long-term methods of contraception	Provision of long acting contraceptives (I)	Women (A) feel confident in their ability to access implants (M) and choose to take up long-acting contraceptives (O)	Self-efficacy
	*“The awareness that we have gained about family planning has also been great … now thanks to the implant I can’t get pregnant while I still have it in. We now try to have a 3–5 year gap between each child.” Group leader_1*		
**CAMO 3**: HEWs are unable to remove implants (C)	No defined intervention (I)	Women (A) worry about their inability to access implant removal (M) and may not choose to take up an implant (O)	Self-efficacy, accessibility, availability, burden, accommodation
	“*R: There are many good things to this project …*. *An improvement I would suggest would be to train us in removals. Women are currently being referred 27 km away. Transport is 30 birr return. This is a burden to them and is hindering the project from reaching higher coverage levels*.” Nurse_1		

### HCW and HEW mechanisms

Mechanisms leading to outcomes of acceptability and adoption of innovations within the integrated service delivery model related to the integrated delivery itself, and to training in integrated delivery. HEWs perceived a decrease in their workload and therefore a reduced work burden given the integration of FP with immunisations, particularly in light of the 45 day immunisation visits, which involved both immunisations and FP counselling and/or provision of MCMs (CIAMO 1). HEWs viewed the integrated delivery of immunisation and FP services positively and felt that providing both services together had more impact than providing them alone (CIAMO 2).

Overall, FP training was viewed positively among HEWs, with the practical elements of using anatomical models for the practice of implant insertions and the clinical coaching of insertions in the community considered particularly helpful. This meant that HEWs were confident in their ability to provide implants for women and that integrated services were provided (CIAMO 3). However, HEWs did not receive training on implant removals and therefore could not provide this service, which left them feeling limited in their ability to provide FP services in general (CAMO 1).

“*There are many good things to this project such as having fully trained extension workers, having the two services integrated. An improvement I would suggest would be to train us in removals. Women are currently being referred 27km away. Transport is 30 birr* [approximately $1 USD] *return. This is a burden to them and is hindering the project from reaching higher coverage levels. If we have 4 or more women we call the health facility workers here to carry out the removals*.” *– Nurse_1*

### Religious leader and male partners mechanims

Religion was the major contextual factor influencing FP acceptance in the study community, and the perceptions and beliefs of religious leaders were powerful. Preventing a child from being born was considered to directly oppose religious principles, particularly Islam. Religious beliefs including that preventing births is *haram* (forbidden) under Islamic law; dying and being buried with an implant in place is *haram*; blood spotting or losing blood that isn’t part of the menstrual cycle is *haram*; and male partners not wanting women to cook during Ramadan whilst using an implant, were seen to hinder acceptability of FP.

Initially, FP acceptability was low among religious leaders and men, and it was thought that this context made it challenging for women to take up and use FP. Recognising this context, the intervention implementers worked with religious leaders to analyse religious texts. This component of the intervention triggered the recognition of the alignment between religious texts and FP in the religious leaders, ultimately resulting in their acceptance of FP (CIAMO 4).

*R: … .. we initially trained religious leaders … … they had different views on whether the religion allowed modern contraceptives or not … .. so we engaged a sheik at the national level, a very supreme sheik who came down and spent three days of just purely going through the Quran linking it to maternal child health, linking the Quran to FP and immunisation and how it is important as religious leaders and husbands and you know. … ..he went through each method and linked it to the Quran and prophet Mohammed and you know said this be acceptable or not, he tracked everything conceptualizing, and you it was a light bulb, and all of them realized we have been teaching the community the wrong thing the whole time …… because they want to just follow the Quran as it is.* Implementing NGO_9

*“R: As religious leader, I use to know that family planning was something bad but we were able to get the other religious leaders, the key Kenyan religious leaders and they taught us about it and even explained to us that it was even used back then in the era of our Prophet .This cleared all the doubts we ever had and we decided to share it to the community.”* Religious leader_3

Similarly, beliefs about FP among male partners were influenced by their religious faith and the views of their religious leaders. If the latter were open about FP, male partners were more willing to support FP use. When religious leaders openly promoted the alignment of FP and religious text and principles, male partners became more supportive of FP, given the respect and trust they held in religious leaders. (CIAMO 5).

*“Some women used to previously access family planning without the knowledge of their husbands but since the religious leaders have now accepted it as a good thing we have now seen the effects trickle down to the husbands. We include them whenever we do community based training. They are now happy as far as I am aware. But they were previously against family planning.” District level administrator_1*

### Post-partum women mechanisms

The one female FP user interviewed, the HEWs, and the community volunteers described factors that influenced women’s decision making around FP use. Such factors included cultural, societal, and religious norms and attitudes towards FP. Support from religious leaders, other community members, and husbands, encouragement from HEWs about the benefits of FP, and knowledge about available FP resources were perceived to be factors that encouraged women to use MCMs. Religious leaders and men finding FP acceptable and in line with their religious beliefs strengthened the community context, which triggered the mechanism of feeling supported by their partners and the wider community, enabling women to confidently accept FP when it was offered to them (CAMO 2).

The training of HEWs on implant insertions was perceived as a positive aspect of the intervention by the one female FP user interviewed and among the HEWs. It was perceived that knowing that long-acting MCMs were available meant that women felt confident in their ability to access these methods (CIAMO 6). Conversely, HEWs’ inability to remove implants presented a problem and HEWs perceived this to mean that women worried about their inability to access implant removals and may choose not to take up an implant (CAMO 3).

### Linking context-mechanism-outcome configurations with implementation related theoretical framework constructs

**Constructs linked to HCW and HEW mechanisms:** empirical mechanisms driving outcomes of the integrated delivery of FP with immunisation services and the training component of the intervention linked to 4 constructs of the TFA (burden, affective attitude, perceived effectiveness and self-efficacy) and 3 constructs of adoption and diffusion of innovations (relative advantage, trialability, and observability).

**Constructs linked to religious leader and male partner mechanisms:** two constructs of the TFA (ethicality and opportunity costs) and 1 construct of the adoption and diffusion of innovations (compatibility) linked to the empirical mechanisms identified in the study as driving outcomes of the integrated delivery of FP and immunisations.

**Constructs linked to post partum women mechanisms**: the major construct identified in this study influencing post-partum women’s uptake of FP within this integrated delivery model was that of sel-effficacy which is a construct of the TFA. Self-efficacy was also negatively influenced by 3 constructs of access which were accessibility, availability and accommodation.

### Revised programme theory

**Fig. 2:**
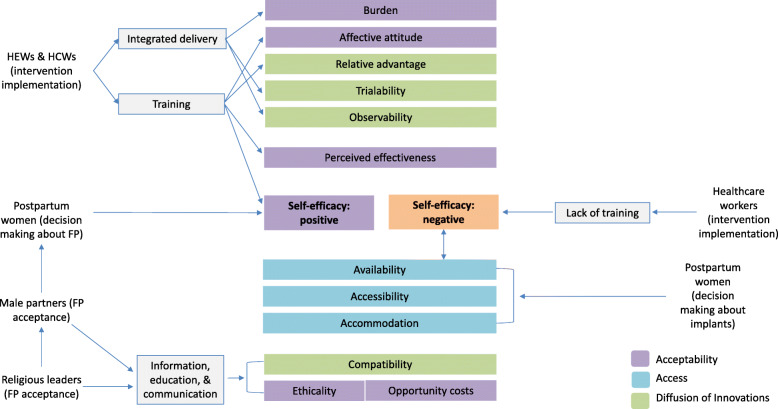
Revised programme theory incorporating middle range mechanisms. Intervention imputs include training on EPI & FP on integrated service delivery, and information, edcuationm and communication about the benefits of FP & socio-cultural alignment. Theoretical constructs within the three different frameworks, shown in different colours, drove actor reactions or decision making about intervention imputs

The CAMOs and CIAMOs were used to develop a revised programme theory (RPT). The RPT represented the intervention integrating FP and immunisation sevices and its key components: training of HEWs and HCWs on EPI and FP integrated service delivery; and information, education, and communication on the benefits of FP and socio-cultural and religious alignment within the community. For each of these components, the RPT represented the empirical mechanisms that drove actors’ responses to the component. The theoretical framework constructs linked to the CAMO/CIAMO configurations of the RPT were then used to build a framework construct linked RPT inclusive of middle range mechanisms (Fig. 2).

## Discussion

In this realist evaluation, we sought to identify key mechanisms driving the implementation of an intervention of integrated FP services and child immunisations in BGRS, Ethiopia. This evaluation contributes to a growing body of literature that seeks to understand uptake of FP when FP services are integrated with other health services [7–9, 26–29]. Recent studies from Rwanda, Zambia, and Ghana have looked specifically at the integration of FP with immunisations services and have found varying levels of success [7–9]. Issues such as inconsistent training for HCWs, poor monitoring systems, and disjointed referral systems have all been cited as barriers to effective integration [7–9]. Central to this evaluation was the exploration of if and how integration worked, for whom and what mechanisms drove FP uptake. There is currently a lack of literature in which context and mechanisms are used to explain intervention integration, and this evaluation offered a unique opportunity to explore this.

### Constructs of implementation related theoretical frameworks as middle range mechanisms

We found empirical mechanisms of integrated delivery of FP with immunisation to link well to constructs of the TFA and the adoption and diffusion of innovations frameworks; and to a lesser extent to the access framework. For example, the perceived reduced work burden of delivering FP and immunisation through household visits increased the acceptability of the intervention for HEWs. The integrated delivery was thereby seen to have a relative advantage over the additional household visits needed to deliver the services seperately. This was linked to both the TFA and adoption and diffusion of innovations frameworks. Although there were some overlaps between links to TFA constructs and adoption and diffusion of innovations framework constructs in terms of HCW and HEW mechanisms, the frameworks were overall complementary. At the community level, the TFA constructs of ethicality and opportunity costs linked well with empirical mechanisms of recognising the alignment between religious texts and FP principles. Compatibility is a similar construct and therefore did not add extra value. Access constructs were linked to the mechanism of feeling unable to access implant removals, which led to negative self-efficacy.

We suggest that the constructs of the TFA, adoption and diffusion of innovations, and access frameworks act as middle-range mechanisms, that is, mechanisms with a relatively high level of abstraction compared to empirical mechanisms. For example, ethicality is a middle-range mechanism, whilst the recognition of alignment with religious texts and FP by religious leaders is an empirical mechanism triggered among religious leaders in a context of strong religious beliefs.

### Middle-range mechanisms

#### Self-efficacy

Was a key construct among health workers and community members. Self-efficacy has been described as ‘an individual’s belief in his or her capacity to execute behaviors necessary to produce specific performance attainments’ [30]. Among HEWs, feelings of self-efficacy were seen to drive motivation for and perceptions of the intervention. HEWs felt confident that they were sharing their workload with their co-workers. HEWs knew that they could carry out their work effectively, as the work was being shared, and this fostered a sense of teamwork among them. Studies that have assessed self-efficacy among health workers have found strong links between feelings of self-efficacy and motivation and have emphasized the links between team work, task-sharing, and self-efficacy [31–33].

**T**he willingness of the HEWs to attend and engage with training was a key contributor to intervention outcomes, as these translated into HEWs feeling high levels of self-efficacy when delivering FP services. However, HEWs were not trained in implant removals and this was perceived to have adversely affected uptake of implants by women. HEWs expressed concerns about women having to travel far distances and incur costs to access larger health facilies where implant removals were carried out. This led to reduced self-efficacy among HEWs. It should be noted that national policy in Ethiopia does not currently task HEWs with implant removals [13] which has resulted in an unmet need of implant removals in the many rural and hard to reach areas [13, 34]. To address this, the Integrated Family Health Programme (IFHP) has attempted to scale-up the availability of trained health professionals who can provide this service, but the programme has yet to be extensively rolled out. The Ethiopia Ministry of Health had begun to pilot the training of select HEWs in implant removals across the country, including in BGRS [34].

At the community level, feelings of self-efficacy were triggered among women when they felt there was community support for FP use, particularly among male partners. This finding is supported by recent literature. A study from Guatemala found that feelings of self-efficacy were negatively impacted by the lack of knowledge about and availability of methods, the fear of side effects and of infertility, and husbands being against FP [35].

#### Relative advantage and burden

HEWs perceived integration of immunisation and FP services to be advantageous. Relative advantages included reduced workloads (burden), and a clear fit with their schedule, which focused on providing FP counselling during post-natal household visits and MCMs during the ‘45 day immunisation’ visit. A recent Cochrane review of integrated interventions found that HCWs may become overloaded or deskilled in integration interventions leading to negative impacts on service provision and health outcomes [1]. However, our findings indicate that teamwork among HEWs, HCWs, and HDA members resulted in manageable workloads and a reduced burden.

The constructs triggered in our evaluation indicate that in order for health workers to perceive the intervention positively, they needed to see how it would be advantageous to them or their clients, and how it would reduce their workload. Studies that have explored the training of community-based health workers have cited manageable workload, organisation of tasks, supportive supervision, adequate supplies and equipment, and respect from the community and the health system as key drivers of successful service delivery [9, 36]. A recent study by Mayhew et al further supports our findings by concluding that structural factors at the health facility level, including issues of staffing and workload in integrated interventions can be mitigated and managed by HCWs themselves [37]. The authors highlight that when HCWs felt agency or power over their own decision-making, they were able to overcome potential challenges of integration [37]. These factors were mentioned by the HEWs interviewed in this evaluation and indicate that while the training they received was important, its effectiveness was dependent on having a supportive work environment that included workload sharing with colleagues, which triggered mechanisms of self-efficacy. This suggests that self-efficacy, relative advantage, and burden might be intrinsically linked, with the perceptions about the advantages of the intervention, and about the ability to share the workload, leading to feelings of confidence among HCWs and HEWs in their ability to deliver the intervention.

#### Ethicality

Acceptability of FP by religious leaders and community members, including men, was a key factor driving wider community acceptability and in turn, influencing women’s decisions around FP. FP acceptance among religious leaders was triggered by their ability to see that FP aligned with their religious beliefs. Knowing that they did not have to adjust or compromise their religious beliefs to support the use of FP is what drove acceptability among religious leaders. The influence of religious leaders on the health seeking behaviours of communities is well documented. A recent study from Nigeria found that women’s attendance at ANC services increased after religious leaders in the community began promoting ANC as an essential component of maternal and child health [38]. In this context, religious leaders had a key role in the delivery of health messages. Similarly, Azmat et al. (2011) determined that religious leaders in Pakistan held a strong influence on communities and that they could play a key role in informing the community about the benefits of FP. FP acceptance among religious leaders in this context was influenced by exposure to messaging and information about FP from medical professionals [39].

Our findings suggest that FP acceptance among religious leaders led to FP acceptance among community members, particularly among men. Ethiopia’s 2016 DHS data indicate that men have more decision-making power than women within couples regarding FP in BGRS compared to almost every other region in the country [23]. Studies from Nigeria and Malawi support the argument that men influence women’s decision-making about FP and that a key component of FP interventions should be male partner education to encourage their support for FP [40–42]. DHS data also indicate that women in BGRS have lower rates of FP use than women in almost every other region in the country [43]. They are also less likely to give birth with skilled birth attendants either in health facilities or at households [43]. This indicates poor links with the formal health system and limited access to health services.

### What this study adds

This evaluation demonstrates the empirical context-linked mechanisms that drove intervention outcomes among HCWs, religious leaders and community members, in the integrated delivery and uptake of FP services. By linking empirical findings to published theories of acceptability, adoption and diffusion of innovations, and accessibility, middle-range mechanisms were identified, that is mechanisms with a higher level of abstraction, which facilitate the cumulation of learnings from this and other evaluations. We identified self-efficacy, burden and relative advantage, and ethicality as particularly important middle-range mechanisms in our study of the integrated delivery of FP and immunisations.

### Limitations

While SSIs were conducted with a wide range of stakeholders who were selected based on the initial programme theory, it is possible that a larger sample size would have yielded data describing additional CIAMOs to those presented in this paper. Also, only one woman was interviewed specifically for her role as an FP user. HDA members and other community volunteers were sometimes also female FP users. While they provided their perspectives as female FP users, they were not interviewed specifically for that role. A larger sample of female FP users and non-users would have yielded more perspectives from these groups.

## Conclusions

In this study, key contextual factors identified were: the predominant use of trained HEWs to deliver FP services at health posts and in communities; a strong belief in values among religious leaders and community members that challenged the use of MCMs; and a lack of support for FP from male partners based on religious values. These contextual factors, combined with intervention components that emphasised the training of HEWs and HCWs on FP counselling and service delivery, the alignment of religious texts with FP concepts, and the use of religious leaders as agents of change, were found to trigger several mechanisms of acceptability, adoption of innovations and access. Key mechanisms included: a perceived relative advantage of integration and increased self-efficacy among HEWs and HCWs; religious leader acceptance of FP; and acceptance of FP among communities and male partners. By linking context and intervention components to the mechanisms they triggered, this evaluation describes how the intervention worked and for whom. By linking our findings to published theories we were able to identify middle-range mechanisms and to develop a revised programme theory that can be applied to the integrated delivery of FP services in similar contexts.

## Supplementary Information

**Additional file 1.**

## Data Availability

The datasets generated and/or analysed during the current study are not publicly available but are available from the corresponding author on reasonable request.

## Abbreviations

A: Actor
BGRS: Benishangul-Gumuz Regional State;
C: Context
CIAMO: Context-Intervention-Actor-Mechanism-Outcome
CAMO: Context-Actor-Mechanism-Outcome
CMO: Context-Mechanism-Outcome
FP: Family Planning
HCW: Health Care Worker
HDA: Health Development Army
HEW: Health Extension Worker
I: Intervention
In: Interviewer
IRC: International Rescue Committee
IUD: Intrauterine Device
M: Mechanism
MCM: Modern Contraceptive Method
O: Outcome
SSI: Semi-Structured Interview.
