# Supplementary material for: What mechanisms drive uptake of family planning when integrated with childhood immunisation in Ethiopia? A realist evaluation
Source: BMC Public Health. 2021 Jan 7;21:99. doi: 10.1186/s12889-020-10114-8 (PMC7791767; doi:10.1186/s12889-020-10114-8)
Supplement: Supplementary file 1 — Additional file 1. [file 12889_2020_10114_MOESM1_ESM.docx]

**Questions for Health Development Army Leaders, Health Extension Workers, health care providers, community leaders:**

Tell me about the integration of immunizations and FP services.

What was your involvement in the project?

What training did you receive for this project?

Do you think the training was good/bad?

Do you think that the project went well (or is going well)?

Why/Why not?

How/Did the intervention affect you directly?

What do you think were the good things about the project?

What do you think were the challenges of the project?

Do you think there is a need for a project like this in this community?

What do you think has changed in terms of availability of FP services?

Have there been awareness campaigns about FP services?

What have the awareness campaigns taught you, and others in the community, about FP services?

*Do you think it makes sense to measure uptake on the same day of counselling, or do women need longer to decide on an FP method?*

*What are some of the social/cultural barriers that women face when accessing FP services?*

*Do you think that the integration project addresses these issues?*

**Additional questions for HEWs**

What training did you receive about this intervention?

Do you have time to promote this intervention?

What are the time restrictions?

Do you prefer to counsel women about one type of FP over another?

Why/Why not?

Do you enjoy being involved in this project?

**Questions for mothers**

Tell me about the integration of immunizations and FP services.

What did you think about this project?

Do you think this was a good project? Why/why not?

Were you accessing FP services before the project started?

Are you accessing FP services now?

If you feel comfortable, can you please tell me what form of FP you are using now?

Why are you using this method?

How has your (male) partner reacted to you receiving FP counselling/services?

How have other members of your social group reacted to you received FP counselling/servicses?

Do you discuss FP with your friends? What do you talk about?

What have the awareness campaigns told you about FP?

What are some things that restrict you from accessing services?

What has changed for you since the beginning of this project?

*For those who accessed services:*

Did you find it easy to access the project?

Were you happy about receiving counselling?

**Questions for male partners**

Tell me about the integration of immunizations and FP services.

What did you think about this project?

Do you think this was a good project? Why/why not?

How have you been involved in the project (i.e. were you part of a male engagement group etc)?

If you feel comfortable, can you please tell me whether your wife (or female partner) uses FP services?

What do you think are some of the barriers women face when accessing FP services?

What have the awareness campaigns told you about FP?

Do you support women accessing FP services?

Why/Why not?
